# Consumption of Key Food Groups by Individuals Consuming Popular Diet Patterns: Mixed Effects of Replacing Foods High in Added Sugar, Sodium, Saturated Fat, and Refined Grains

**DOI:** 10.3390/nu14245226

**Published:** 2022-12-08

**Authors:** Sarah Rowe, Avonti Basak Tukun, LuAnn K. Johnson, David C. Love, Martha A. Belury, Zach Conrad

**Affiliations:** 1 College of Arts & Sciences, William & Mary, Williamsburg, VA 23185, USA; 2 Program of Human Nutrition, The Ohio State University, Columbus, OH 43221, USA; 3 Independent Contractor, Warren, MN 56762, USA; 4 Johns Hopkins Center for a Livable Future, Johns Hopkins University, Baltimore, MD 21202, USA; 5 Department of Environmental Health and Engineering, Johns Hopkins Bloomberg School of Public Health, Baltimore, MD 21205, USA; 6 Department of Kinesiology, William & Mary, Williamsburg, VA 23185, USA; 7 Global Research Institute, William & Mary, Williamsburg, VA 23185, USA

**Keywords:** NHANES, popular diet, fad diet, diet pattern, vegetarian, restricted carbohydrate

## Abstract

Adults in the United States are increasingly following ‘popular’ diet patterns that restrict food groups, macronutrients, or eating time. However, the intake of food groups associated with these diet patterns has not been well characterized. The objectives of this study were to (1) characterize the mean intake of food groups among consumers of popular diet patterns in the US, and (2) model the effect of targeted food substitutions on the intake of food groups. Data were acquired from the National Health and Nutrition Examination Survey, 2005–2018 (n = 34,411). A diet model was developed to assess the effects of replacing one serving each of foods highest in added sugar, sodium, saturated fat, and refined grains with healthy alternatives on the intake of key food groups for each diet pattern. Modeled replacement resulted in increased intake of fruit and whole grains and decreased intake of dairy for most diet patterns, while the effects on the intake of vegetables, protein foods, and oils were variable across diet patterns. The complexity of the natural eating environment, in which many people consume mixed dishes that include both healthy and less healthy ingredients, produces a challenge for health professionals when providing dietary counseling. Nevertheless, this substitution approach may help improve adherence to dietary guidelines, especially if used as a steppingstone for further dietary improvement.

## 1. Introduction

Less than 3% of adults in the United States (US) consume a diet that is consistent with dietary recommendations [1]. Suboptimal diet quality is the largest preventable risk factor for death in the US, contributing to approximately 500,000 deaths per year, largely from cardiometabolic diseases including heart disease, stroke, and diabetes [2]. Diet-related disability accounts for over 10% of total life years, representing the third largest preventable risk factor for morbidity [2]. Over half of Americans suffer from at least one diet-related chronic condition [3].

Despite modest improvements in diet quality over the past 20 years, adherence to the Dietary Guidelines for Americans (DGA) remains far below optimal [4]. These guidelines encourage limiting consumption of foods high in added sugar, sodium, saturated fat, and refined grains in favor of healthier alternatives such as fruits, vegetables, nuts and seeds, whole grains, and sources of unsaturated fats [5]. Rather than choosing to follow these principles when making food choices, adults increasingly following ‘popular diets’ [6] that restrict food groups, macronutrients, or eating time [7]. These include plant-based, low carbohydrate, and intermittent fasting diet patterns [6]. National consumer surveys show that over half of US adults reported following at least one of these diet patterns in 2022, an increase of 13 percentage points over the previous year [6].

Nationally representative dietary data collected from multiple 24 h recalls show that 44% of adults followed at least one popular diet pattern (vegetarian, pescetarian, low grain, high protein, restricted carbohydrate, or time-restricted) on a given day and nearly 10% followed more than one diet pattern [8]. Protecting long-term health consistently remains one of the top motivations for adopting popular diet patterns, followed closely by weight loss [6], but the evidence to support their health benefits remains sparse [7]. Conrad et al. reported that the diet quality of many popular diet patterns as measured by the Healthy Eating Index (HEI) was far below optimal (overall score not exceeding 65 out of 100) [8], which warrants increased efforts to improve dietary intake among those consuming popular diets.

However, public health guidance regarding foods groups to encourage or to limit may not always translate well to actionable clinical counseling because individuals often consume foods as mixed dishes that include multiple ingredients, so following these recommendations may have unexpected effects on the intake of target food groups. Considering the substantial impact of individual food groups on reducing disease risk [9,10] and the high prevalence of popular diet patterns [6,8], a better understanding of how targeted food substitutions can impact the intake of recommended food groups is needed in order to support actionable clinical counseling for people following popular diet patterns.

To fill this research gap, the present study (1) assessed the mean intake of food groups for several popular diet patterns in the US using data from a nationally representative sample, then (2) modeled the effects of targeted food substitutions that align with DGA recommendations on the mean intake of recommended food groups.

## 2. Materials and Methods

### 2.1. Data Acquisition

Data on daily intake of foods, nutrient intake from foods and supplements, and sociodemographic characteristics were obtained from the National Health and Nutrition Examination Survey (NHANES), 2005–2018. NHANES collects data from approximately 5000 non-institutionalized participants per year using a multi-stage, clustered, stratified sampling design [11]. Data are collected by trained staff using in-person surveys, laboratory tests, and physical examinations. Some population groups are oversampled to increase reliability and precision for subgroup analysis [12]. Trained interviewers collect dietary data using the computer-assisted Automated Multiple Pass Method to increase reliability and validity and minimize respondent burden [13,14]. A second recall is administered by telephone 3–10 days later for approximately 80% of participants [15]. The salt adjustment was appropriately removed from dietary data collected from 2005–2008 to allow for consistent measurement of sodium intake across all data years [16]. Many foods reported to be consumed by NHANES participants are mixed dishes that include multiple food groups, such as lasagna, which can include grains (usually refined), meat (such as ground beef), vegetables (tomato sauce), dairy (cheese), and vegetable oils. The amount of each food group in each NHANES food was estimated using the Food Patterns Equivalents Database (FPED) [17]. The present study is a secondary analysis of de-identified and publicly available data and was exempted from human studies ethical review by the Institutional Review Board at William and Mary. Pre-registration for this study can be found elsewhere [18].

### 2.2. Current Dietary Intake

The National Cancer Institute’s (NCI) usual intake methodology was used to estimate current intake of food groups, kcal, and nutrients. This methodology estimates within-person variation of the entire sample using data from multiple 24 h recalls collected from most participants [19]. The SAS macros MIXTRAN (v2.21) and INDIVINT (v2.3) were used to estimate individual-level predicted intake, and the macros NLMIXED_UNIVARIATE (v1.2), NLMIXED_BIVARIATE (v1.2), and PREDICT_INTAKE_DENSITY (v1.2) were used to estimate nutrient densities (e.g., percent energy from carbohydrate) [20,21,22].

### 2.3. Diet Pattern Categorization

Data on usual intake of foods and nutrients were used to categorize diet patterns for each participant into food group-restricted (vegetarian, pescatarian, low grain), macronutrient-restricted (high protein, restricted carbohydrate), and time-restricted, with the total sample denoted as the general population. Information on popular diet patterns from published literature was used to construct these categories [6,7,23,24,25] and is described in Table S1. Data on daily intake of food groups from FPED were used to categorize food group-restricted diet patterns; data on daily intake of nutrients from NHANES were used to categorize macronutrient-restricted diet patterns; and data from NHANES individual food files, which provide information on the time of each eating occasion for each participant, were used to categorize the time-restricted diet pattern. Diet patterns were not necessarily mutually exclusive (e.g., some participants may consume restricted carbohydrate and high protein diet patterns). Non-consumers were identified if they did not consume a given food group on both days of recall, because the NCI methodology does not predict non-zero intake.

### 2.4. Food Categories and Serving Sizes

The food categories used in the Food and Nutrient Database for Dietary Studies (FNDDS) [26] and FPED [27] were used to categorize each food and beverage consumed by each participant on the first day of dietary recall into one of 89 categories (hereafter, food categories). For example, FNDDS was used to identify fruit juices and FPED was used to further disaggregate into 100% juice and juice with added sugar (Table S2). Data on the weight of each food consumed (in grams), as well as their content of kcal and nutrients, were acquired from NHANES files, and data on the amount of each food group within in each food were acquired from FPED files. These data were used to estimate the average servings sizes of each food category and the average amount of added sugar, sodium, saturated fat, refined grains, kcal, and each food group present per serving of each food category, as described below.

For each food category, serving sizes were estimated by taking the mean weight of each food within each category for each eating occasion for the entire sample. For example, there were 115 types of 100% juices that were reported to have been consumed on 9550 occasions, and the average amount (by weight) consumed at each eating occasion was used as the serving size for this food category. For packaged foods and beverages, estimated serving sizes were similar to serving sizes listed on the Nutrition Facts Panel of these products [28], and for non-packaged foods, estimated serving sizes were similar to serving size estimates provided by FPED documentation [27]. The amount of added sugar, sodium, saturated fat, refined grains, kcal, and each food group per serving of each food category was estimated by calculating the average amount of these food components present per gram of each food for each food category and then multiplying by the result by the mean serving size (in gram weight) of each food category.

### 2.5. Target Foods and Alternative Foods

Food categories to be removed during modeling (target foods) were those that contributed the greatest daily amount of added sugar, sodium, saturated fat, and refined grains for each diet pattern. Food categories to be added during modeling (alternative foods) were identified using the following conditions: (1) did not violate the definition of each diet pattern (e.g., poultry was not used as an alternative food for the pescatarian diet pattern), (2) represented a sensible dietary substitution that individuals may make in real world situations, as determined through consultation with multiple Registered Dietitian Nutritionists (RDN) as documented by Conrad et al. (2022) (e.g., dishes were replaced with dishes, beverages with beverages, snacks with snacks, and desserts with desserts) [8], and (3) of the remaining options it was consumed in the greatest amount.

### 2.6. Diet Modeling

A diet model was developed to assess changes in intake of DGA food groups (whole fruit; fruit juice; dark green vegetables; red and orange vegetables; starchy vegetables; other vegetables; beans, peas, and legumes; refined grains; whole grains; meat; poultry, seafood; eggs; soy products; nuts and seeds; dairy; and oils) if one serving of each target food (i.e., food categories highest in added sugar, sodium, saturated fat, and refined grains) were replaced with one serving of alternative foods (4 servings total). For each substitution, the amount of each food group and kcal in each serving of the target food was extracted from each participant’s diet pattern, and the amount of each food group and kcal contained within each serving of the alternative food was added to each participant’s diet pattern. To allow for discretionary intake, which may increase achievability for some individuals, the model only performed replacements if a participant consumed ≥1 serving of the target food. This modeling scenario is consistent with DGA 2020–2025 recommendations to limit consumption of these food components by making nutrient-dense food substitutions [5].

### 2.7. Statistical Analyses

Intake of food groups and kcal were estimated at baseline and after each modeled replacement. All values were adjusted for age, gender, and survey wave using linear regression models. Differences between current and modeled intakes for each diet pattern were assessed using paired Wald tests with a two-tailed distribution at *p* < 0.05. NHANES design variables and survey weights were used to account for the multistage probability sampling design and to produce nationally representative estimates. SAS 9.4 (SAS Institute; Cary, NC, USA) was used to estimate usual intakes using the NCI macros, and Stata 16.1 (StataCorp; College Station, TX, USA) was used for data management and final analyses.

## 3. Results

### 3.1. Participant Characteristics

Dietary data were collected from a total of 61,682 participants from 2005–2018. Participants were excluded from the analysis if they were <20 years (n = 26,375), provided unreliable or incomplete dietary information (as determined by NHANES staff), or were pregnant or breastfeeding (n = 896). A total of 34,411 participants were included in the final sample. 

Participants who were categorized into food group-restricted diet patterns were majority female (64–69%) and non-Hispanic white (61–70%) with a mean age of 45–50 years (Table 1). Most had attained at least some college education (59–68%) and had income-to-poverty ratios ≥2.8.

Most participants that were categorized into macronutrient-restricted diet patterns characterized as high protein or restricted carbohydrate were male (93% and 56%, respectively) and non-Hispanic white (66% and 73%, respectively; Table 1). Compared to individuals that followed a high protein diet pattern, those that followed a restricted carbohydrate diet pattern were older (48 years vs. 40 years), had higher educational attainment (66% completed some college vs. 56%), and had higher income-to-poverty ratios (3.3 vs. 2.6). Participants categorized into the time-restricted diet pattern had a mean age of 43 years, and approximately one-half were female (52%), completed at least some college (48%), and were non-Hispanic white (53%).

### 3.2. Foods and Beverages Highest in Added Sugar, Sodium, Saturated Fat, and Refined Grains

Food sources of added sugar, sodium, saturated fat, and refined grains among individuals that followed popular diet patterns is described in Table S3 and reported in detail elsewhere [8]. Briefly, soft drinks with added sugar provided the greatest contribution to daily intake of added sugar for most diet patterns (16–36% of daily intake), and poultry dishes or pizza were the largest contributors to daily intake of sodium for most diet patterns (6.1–10% of daily intake). The greatest contributor to daily intake of saturated fat for most diet patterns was cheese (8.7–12.3%). Yeast bread provided the greatest contribution to refined grain intake for all diet patterns (20–25%).

### 3.3. Modeled Changes in Food Intake for the General Population and Food Group-Restricted Diet Patterns

**Figure 1:**
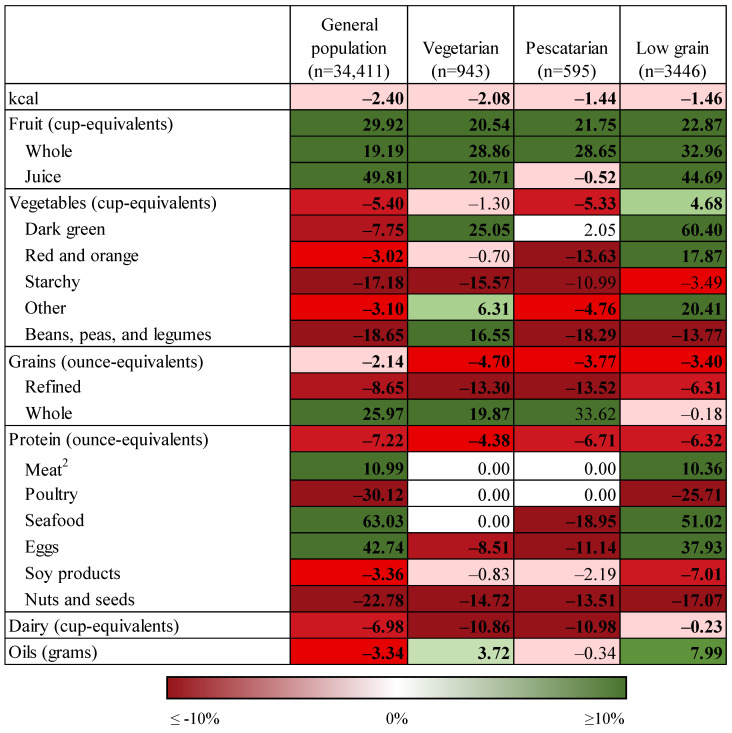
Percent change in intake of food groups under conditions of modeled food replacements ^1^ for food group-restricted diet patterns and the general population. ^1^ One serving substitution of foods highest in added sugar, sodium, and saturated fat with alternative foods (four servings total). ^2^ Does not include cured meat and organ meat.

Figure 1 displays the results of modeled food substitutions for the general population and food group-restricted diet patterns (vegetarian, pescatarian, and low grain) as percent changes from current intake, and Tables S4 and S5 display these results as mean intakes. Modeled replacement of one serving each of foods highest in added sugar, sodium, saturated fat, and refined grains (4 servings total) resulted in decreased intake of kcal by 1–2% for each diet pattern (23–50 kcal). Modeled intake of total fruit increased by 21–30% for each diet pattern (0.2–0.3 cup-equivalents), and fruit juice accounted for the largest share of this increase in the general population (0.14 cup-equivalents) and low grain diet patterns (0.11 cup-equivalents) while whole fruit accounted for the largest share of this increase in the vegetarian (0.23 cup-equivalents) and pescetarian (0.24 cup-equivalents) diet patterns. Total vegetable intake increased for the low grain diet pattern only (5%, 0.10 cup-equivalents), which was driven by increased intake of dark green vegetables (0.11 cup-equivalents), red and orange vegetables (0.07 cup-equivalents), and other vegetables (0.12 cup-equivalents). Among the general population and pescatarian diet pattern, modeled intake of total vegetables decreased by 5% (0.01–0.02 cup-equivalents) and intake of most vegetable subgroups decreased by 3–19%. No change in total vegetable intake was observed in the vegetarian diet pattern due to the mixed effects across subgroups: increased intake of dark green vegetables (0.05 cup-equivalents), other vegetables (0.04 cup-equivalents), and beans, peas, and legumes (0.03 cup-equivalents), and decreased intake of starchy vegetables (0.06 cup-equivalents).

Modeled grain intake decreased for the general population and all food group-restricted diet patterns (Figure 1, Tables S4 and S5) by 2–5% (0.1–0.3 cup-equivalents), which was driven by a decrease in refined grain intake of 6–14% (0.2–0.7 cup-equivalents) and an increase in whole grain intake by 20–34% (0.0–0.29 cup-equivalents). Total protein food intake decreased for all diet patterns by 4–7% (0.07–0.27 cup-equivalents). Among the general population and low grain diet pattern, modeled intake of meat, seafood, and eggs increased (0.11–0.25 cup-equivalents) while intake of poultry, soy products, and nuts and seeds decreased (0.00–0.32 cup-equivalents). Among the vegetarian and pescatarian diet patterns, modeled intake of seafood (pescetarian diet pattern only), eggs, and nuts and seeds decreased by 9–19%. Modeled dairy intake decreased by up to 11% (0.1 cup-equivalents) for all diet patterns. Modeled intake of oils decreased by 3% (0.9 g) for the general population and increased by 4–8% (0.9–1.7 g) for the vegetarian and low grain diet patterns.

### 3.4. Modeled Changes in Food Intake for Macronutrient-Restricted Diet Patterns and Time-Restricted Diet Patterns

**Figure 2:**
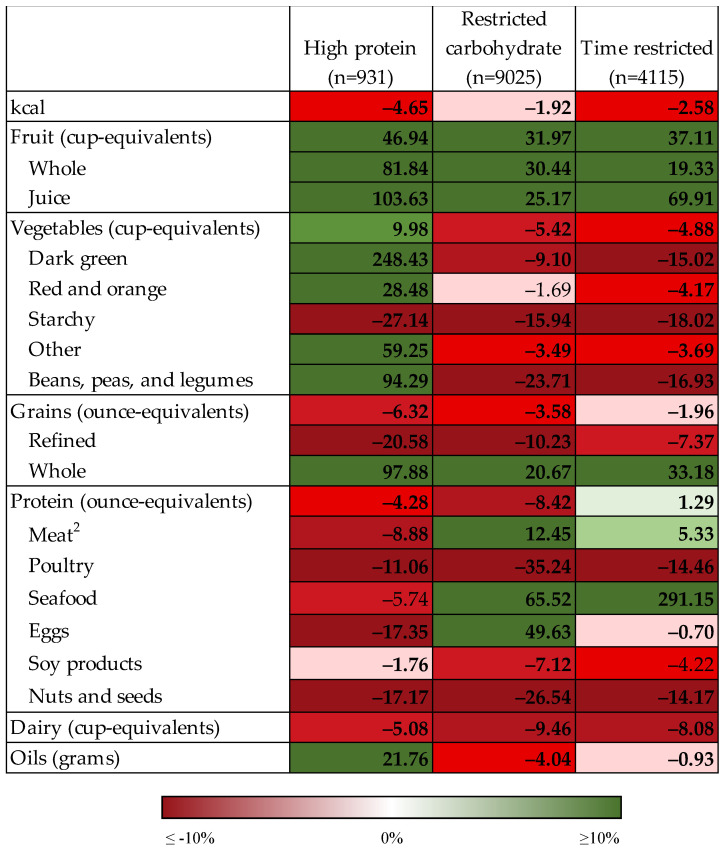
Percent change in intake of food groups under conditions of modeled food replacements ^1^ for macronutrient-restricted diet patterns and time-restricted diet pattern. Bolded text indicates statistically significant difference between current and modeled intake at *p* < 0.05 using paired Wald tests. ^1^ One serving substitution of foods highest in added sugar, sodium, and saturated fat with alternative foods (four servings total). ^2^ Does not include cured meat and organ meat.

Figure 2 displays the results of modeled food substitutions for the macronutrient-restricted diet patterns (high protein and restricted carbohydrate) and time-restricted diet pattern as percent changes from current intake, and Tables S6 and S7 display these results as mean intakes. Modeled substitution of one serving each of foods highest in added sugars, sodium, saturated fat, and refined grains (4 servings total) resulted in decreased intake of kcal by 2–5% for each diet pattern (41–170 kcal). Modeled total fruit intake for each of these diet patterns increased by 32–47% (0.25–0.39 cup-equivalents). The largest portion of this increase was attributable to fruit juice for the high protein (0.40 cup-equivalents) and time restricted (0.18 cup-equivalents) diet patterns and attributable to whole fruit for the restricted carbohydrate diet pattern (0.17 cup-equivalents). Mean total fruit intake was greatest for the high protein diet pattern both before (1.09 cup-equivalents) and after modeled replacement (1.48 cup-equivalents). Modeled intake of total vegetables decreased for the time restricted and restricted carbohydrate diet patterns by 5% (0.07–0.09 cup-equivalents), driven by reductions of 3–24% in most vegetable subgroups. In contrast, modeled intake of all vegetable subgroups increased for the high protein diet pattern by 10–248% (0.13–0.38 cup-equivalents), except for total starchy vegetables which decreased by 27% (0.14 cup-equivalents).

Modeled total grain intake decreased by 2–6% (0.11–0.62 ounce-equivalents) for the time restricted and macronutrient-restricted diet patterns (Figure 2, Tables S6 and S7), which was driven by a decrease in refined grain intake of 7–21% (0.38–1.78 ounce-equivalents) and an increase in whole grain intake of 21–98% (0.16–0.92 ounce-equivalents). The greatest mean whole grain intake both before (0.95 ounce-equivalents) and after modeled replacement (1.87 ounce-equivalents) was observed for the high protein diet pattern. Modeled total protein intake decreased for the high protein and restricted carbohydrate diet patterns by 4–8% (0.22–0.37 ounce-equivalents) but increased for the time-restricted diet pattern by 1% (0.05 ounce-equivalents). For the high protein diet pattern, the modeled intake of all protein subgroups decreased by 2–17% (up to 0.17 ounce-equivalents). Among the restricted carbohydrate and time-restricted diet patterns, modeled intake decreased for poultry, soy products (restricted carbohydrate only), and nuts and seeds by 7–35%; conversely, modeled intake of meat, eggs (restricted carbohydrate only), and seafood increased by 5–291% for these diet patterns. Modeled dairy intake decreased for all diets patterns by 5–9% (0.10–0.15 cup-equivalents). Modeled oil consumption increased by 22% (7.7 g) for the high protein diet pattern but decreased by 1–4% (0.22–1.11 g) for the restricted carbohydrate and time-restricted diet patterns.

## 4. Discussion

This nationally representative study demonstrated that implementing key DGA 2020–2025 recommendations had diverse effects on food group consumption across popular diet patterns. Modeled food replacement led to increased intake of fruit and whole grains and decreased intake of starchy vegetables, nuts and seeds, dairy, and kcal across most or all diet patterns. The effects of the modeled replacement on most vegetable and protein subcategories, as well as oils, were heterogeneous both within and across diet patterns. These results demonstrate the limitations of food-based dietary guidance, given that many foods are consumed as mixed dishes that include favorable and unfavorable ingredients.

Dietary patterns have received increasingly greater research attention for understanding the dietary factors underlying morbidity and mortality [29,30,31]. Accordingly, building healthy dietary patterns has been a central focus of federal dietary guidance since 2015 [32,33] and has received increasing attention from major public health institutions [34]. To improve the quality of diet patterns, the 2020–2025 DGA recommends increasing consumption of fruits, vegetables, whole grains, nuts and seeds, and oils while limiting consumption of added sugar, sodium, saturated fat, and refined grains [35]. Adherence to these recommendations is associated with reduced risk of mortality from all causes, cardiovascular disease, and cancer [29,30,36], and has been attributed to greater intake of key food groups [9,37,38]. For example, Chen et al. (2019) demonstrated that dietary patterns characterized by higher intake of vegetables, fruit, and legumes, and lower intake of red meat, were associated with 21,000 fewer disability-adjusted life years [39]. Although several studies have evaluated the intake of food groups among vegetarian diet patterns [40,41], this research framework has not often been applied to other popular diet patterns despite their large and growing prevalence in the US [6,42]. Recently, Conrad et al. (2022) [8] demonstrated that modeled food substitutions based on DGA 2020–2025 guidance led to only modest improvements in the diet quality of popular diet patterns [8], but the impacts on specific food groups were not evaluated. The present study addressed this research gap by demonstrating that the modeled implementation of federal dietary guidance can have mixed effects on consumption of different food groups across popular diet patterns, which has implications for chronic disease risk.

As foods highest in components to limit (added sugar, sodium, saturated fat, and refined grains) are often consumed as part of mixed dishes that also contain components to encourage (e.g., vegetables and oils), modeled food substitutions can have mixed effects on consumption of target food groups. Rehm and Drewnowski (2019) showed that modeled replacement of dairy foods with foods high in mono- and polyunsaturated fats resulted in a 35% reduction in kcal intake but also lower intake of calcium (29%), vitamin D (49%), vitamin A (26%), riboflavin (18%), niacin (3%), and vitamin B12 (29%), and increased intake of added sugar (22%) [43]. Others showed that replacement of all solid breakfast foods with ready-to-eat cereals led to decreased intake of solid fats (11%) as well as increased intake of whole grains (85%), fiber (14%), vitamin D (14%), iron (55%), and folic acid (105%), but also increased intake of added sugar (5%) [44]. Similarly, modeled substitution of tree nuts for other snack foods demonstrated an increase in overall diet quality score of 9 points out of 100, as measured by the Healthy Eating Index-2010, but this was accompanied by decreased intake of total vegetables, total fruit, whole fruit, whole grains, and dairy; and when only snacks not consisting of non-starchy vegetables, whole fruit, or whole grains were replaced, most of these negative changes were eliminated [45]. These complexities emphasize the importance of tailoring dietary advice to meet the needs of each individual in clinical settings in order to avoid unintended consequences.

The present study builds off previous research that demonstrates the value of encouraging consumers to make small rather than dramatic changes to their diet for health improvement [46,47]. Small and sustained surfeits of energy intake can result in long-term weight gain, suggesting that small shifts toward more nutrient-dense food choices can help weight management. Such an approach may also be more sustainable for individuals to maintain since fewer lifestyle changes are required [46,47]. This study evaluated one possible method of implementing small dietary changes for individuals following popular diet patterns, in line with key DGA 2020–2025 recommendations. This study found that simple food and beverage substitutions could produce beneficial changes in the intake of some target food groups, although the majority of these changes were modest (for example, 19% increase in whole fruit intake for the general population). Intake of other target food groups decreased under the same conditions, and these outcomes varied by diet pattern (for example, 5% decrease in total vegetable intake for the restricted carbohydrate diet pattern).

The magnitude of dietary change was greatest for the macronutrient restricted and time-restricted diet patterns for total fruit (up to 0.39 cup-equivalents) and whole grains (up to 0.92 oz-equivalents). Others have demonstrated that each additional 100 g increase in daily intake of fruit is associated with a 6–14% reduced risk of coronary heart disease, stroke, and all-cause mortality [9,48], and Micha et al. (2017) reported that each additional 50 g increase in daily intake of whole grams is associated with a 3–12% reduced risk of cardiovascular disease and diabetes [9]. These findings show that even small dietary changes can yield meaningful health gains if sustained over time and can yield even larger health gains for every incremental improvement in dietary intake. These results provide a valuable resource to health professionals when counseling their clients about practical dietary changes to increase consumption of food groups to align with federal dietary guidelines.

This study has several limitations. All self-reported dietary data may be influenced by social desirability bias, which motivates respondents to misreport their dietary intake in order to seem healthier. This can contribute to misclassification bias, in which respondents are assigned to an incorrect diet pattern. However, the data collected from 24 h recalls still remain a useful tool for comparing diet patterns among large groups. In addition, although healthy alternatives were carefully selected for the modeling phase, these replacement foods may not reflect the substitutions that every consumer would choose, and different food substitutions may yield different results. In addition, substitutions were performed on the basis of servings rather than grams or kcal to reflect the units in which most people make food substitutions, so using another unit of measurement to make substitutions may produce different results. Some popular diet patterns such as vegan and grain-free could not be included in the analysis as a consequence of small sample sizes in the NHANES data, although this may be a viable subject for future exploration as the prevalence of these diet patterns may be increasing based on self-reports [6].

This study also has several strengths. To the best of the authors’ knowledge, this study is the first to evaluate the effect of modeled food substitutions on the intake of DGA-recommended food groups among a nationally representative sample of US adults consuming popular diet patterns. Dietary data were collected from nearly 35,000 participants over a 14-year period which provided a sufficient sample size for analysis even of diet patterns with low prevalence. Diet patterns were established based on self-reported dietary data from 24 h recalls rather than self-categorized diet patterns, which provides a less biased way to estimate dietary intake. For example, self-reported vegetarians often report meat intake [49], and post hoc analyses revealed that fewer than 50% of individuals intending to follow a low carbohydrate diet are classified as such based on repeated 24 h recalls. The modeling component of this study directly addressed the DGA 2020–2025 recommendation to replace foods high in added sugar, sodium, saturated fat, and refined grains with healthy alternatives and was designed to mimic real world decision-making in several ways: all substitutions adhered to the definitions of each diet pattern (i.e., poultry was not used as an alternative food in the pescatarian diet pattern), reflected the foods and beverages consumed in the greatest quantity, were selected with input from multiple Registered Dietitian Nutritionists to ensure applicability for counseling settings, and allowed for discretionary intake of target foods rather than outright elimination from the diet. Finally, substitutions were made based on serving sizes in order to reflect the units in which most consumers make food decisions, rather than on the basis of mass quantity or kcal.

## 5. Conclusions

Among consumers of popular diet patterns, modeled replacement of foods highest in added sugar, sodium, saturated fat, and refined grains led to favorable but modest effects on the intake of some key food groups (e.g., fruit and whole grains) but mixed effects on others (e.g., vegetables and protein foods). The complexity of the natural eating environment, which includes mixed dishes comprised of both foods to limit and foods to encourage, presents a challenge for health professionals seeking to implement federal dietary guidance. Nevertheless, the benefits of modeled replacement on some target food groups in addition to evidence demonstrating the importance of diet to health outcomes suggest that this approach may help improve adherence to dietary guidelines especially if used as a steppingstone for further dietary improvement.

## Figures and Tables

**Table 1:** Characteristics of study participants, 2005–2018 (n = 34,411).

Characteristic	General Population ^1^ (n = 34,411)		Food Group Restricted						Macronutrient Restricted				Time Restricted ^7^ (n = 4115)	
			Vegetarian ^2^ (n = 943)		Pescetarian ^3^ (n = 595)		Low Grain ^4^ (n = 3446)		High Protein ^5^ (n = 931)		Restricted Carbohydrate ^6^ (n = 9025)			
	Mean or Percent (95% CI) ^8^													
Percent of population	100.0		2.6	(2.3–2.9)	1.7	(1.4–1.9)	10.2	(9.7–10.7)	2.7	(2.4–3.0)	28.7	(27.8–29.6)	9.2	(8.7–9.7)
Age, years	47.8	(47.3–48.3)	45.0	(43.3–46.6)	50.3	(48.4–52.3)	50.1	(49.4–50.9)	39.8	(38.7–40.9)	47.8	(47.2–48.3)	42.6	(41.7–43.5)
Female	51.0	(50.3–51.6)	64.0	(59.8–68.1)	65.9	(60.0–71.2)	69.5	(67.3–71.7)	6.6	(4.5–9.7)	44.0	(42.6–45.4)	51.9	(49.6–54.2)
At least some college	60.5	(58.8–62.2)	68.4	(63.6–73)	65.5	(59.9–70.7)	59.2	(56.4–62.0)	56.2	(51.7–60.7)	65.7	(63.7–67.7)	48.1	(45.2–50.9)
Income-to-poverty ratio	3.0	(2.9–3.1)	3.0	(2.8–3.2)	3.3	(3.0–3.5)	2.8	(2.7–2.9)	2.6	(2.4–2.8)	3.3	(3.2–3.4)	2.4	(2.3–2.5)
Race/ethnicity														
Non-Hispanic white	67.3	(64.7–69.7)	61.2	(55.7–66.4)	63.9	(58–69.4)	69.5	(66.1–72.6)	65.6	(60.8–70.2)	73.4	(71.0–75.7)	52.5	(48.7–56.4)
Non-Hispanic black	11.3	(10.0–12.8)	5.3	(3.9–7.1)	10.2	(8.3–12.5)	14.9	(12.9–17.3)	13.0	(10.3–16.2)	10.7	(9.4–12.1)	21.9	(19.1–24.9)
Other	21.4	(19.6–23.3)	33.5	(28.6–38.8)	25.9	(21.1–31.4)	15.6	(13.7–17.7)	21.4	(17.8–25.4)	15.9	(14.3–17.7)	25.6	(22.9–28.5)

Adapted with permission from Conrad et al. (2022). Quality of popular diet patterns in the United States: evaluating the effect of substitutions for foods high in added sugar, sodium, saturated fat, and refined grains. *Current Developments in Nutrition*, 12:nzac119. Sample sizes are unweighted. ^1^ All participants that met inclusion criteria, including those in each diet category as well as those not categorized into diet categories. ^2^ Zero intake of meat, poultry, and seafood. ^3^ Zero intake of meat and poultry and >0 ounce-equivalents of seafood. ^4^ ≤10th percentile of total grain intake. ^5^ ≥30% kcal from protein. ^6^ Less than 45% kcal from carbohydrate. ^7^ Less than or equal to 12 h food and beverage fast. ^8^ Age and income-to-poverty ratio values are means, and the values for all other characteristics are percentages. All values are adjusted for survey weights and multistage survey design.

## Data Availability

Data described in this manuscript, code book, and analytic code will be made publicly and freely available without restriction at https://archive.org/details/osf-registrations-z3fme-v1. This study was pre-registered at the Center for Open Science, Open Science Framework at https://archive.org/details/osf-registrations-z3fme-v1 (accessed on 3 December 2022).

## Supplementary Materials

The following supporting information can be downloaded at: https://www.mdpi.com/article/10.3390/nu14245226/s1, Table S1: Diet categorization; Table S2: Food and beverage categorization; Table S3: Foods and beverages highest in added sugar, sodium, saturated fat, and refined grains, by diet pattern, 2005–2018 (n = 34,411); Table S4: Mean intake of food groups under conditions of modeled food replacements for the general population and vegetarian diet pattern; Table S5: Mean intake of food groups under conditions of modeled food replacements for pescatarian and low grain diet patterns; Table S6: Mean intake of food groups under conditions of modeled food replacements for high protein and restricted carbohydrate diet patterns; Table S7: Mean intake of food groups under conditions of modeled food replacements for time-restricted diet pattern.
